# Impacts of Climate Change on Habitat Suitability and Landscape Connectivity of the Amur Tiger in the Sino-Russian Transboundary Region

**DOI:** 10.3390/ani15172466

**Published:** 2025-08-22

**Authors:** Die Wang, Wen Li, Nichun Guo, Chunwang Li

**Affiliations:** 1 College of Resources and Environment, Anhui Agricultural University, Hefei 230036, China; 15587679587@163.com (D.W.);; 2 Key Laboratory of Animal Ecology and Conservation Biology, Institute of Zoology, Chinese Academy of Sciences, Beijing 100101, China; 3 College of Life Sciences, Hebei University, Baoding 071002, China; 4 College of Life Sciences, University of Chinese Academy of Sciences, Beijing 100049, China

**Keywords:** felidae, ecological corridor, habitat suitability, species distribution models, transborder conservation

## Abstract

The Amur tiger (*Panthera tigris altaica*) is a flagship and umbrella species of forest ecosystems in northeastern Asia. Climate change is profoundly and irreversibly affecting wildlife habitat suitability, especially for large mammals. To effectively protect the Amur tiger, it is necessary to understand the impact of climate change on the quality and the connectivity of its habitat. We used the species distribution models combined with the latest Shared Socioeconomic Pathway (SSP) climate scenarios to predict current and future changes in habitats and corridors. We found the following: (1) The total area of the Amur tiger’s suitable habitat currently amounts to approximately 4941.94 km^2^, accounting for 27.64% of the study area represented by two adjacent national parks. Among these habitats, the highly suitable areas are mainly located on the both sides of the Sino-Russian border. The landscape connectivity remains relatively stable, and the degree of fragmentation in highly suitable habitats is low. (2) Although the suitable habitat of the Amur tiger shows an expansion trend under most climate scenarios (excluding SSP3-7.0), the area of suitable habitat within the entire study region does not increase significantly. Therefore, we should implement conservation measures to facilitate the continued expansion of suitable habitat for the Amur tiger. The quantity and length of landscape connectivity corridors are expected to vary in response to changes in core habitat patches, while the centroid of highly suitable habitats is also expected to shift to different extents. In such circumstances, new ecological corridors need to be constructed, while existing natural ecological corridors should be preserved.

## Simple Summary

The Amur tiger (*Panthera tigris altaica*), a flagship and umbrella species in the forests of northeastern Asia, faces significant habitat challenges due to climate change. To assess these impacts, species distribution models integrated with Shared Socioeconomic Pathway (SSP) climate scenarios were used to evaluate present and future habitat suitability and connectivity. Projections under most future climate scenarios indicate an overall expansion of suitable habitats, with habitat centroids shifting and landscape corridors requiring adaptation. To ensure the long-term conservation of the Amur tiger, it is essential to preserve existing ecological corridors, construct new ones, and expand the species’ habitat range in response to climate-induced changes.

## 1. Introduction

The “State of the Global Climate in 2024” report, released by the World Meteorological Organization, stated that the global average temperature had risen by 1.55 °C above pre-industrial levels [1]. Signs of human-induced climate change are becoming increasingly evident, and the world is undergoing unprecedented changes in the climate system [1]. Climate change affects all levels of biodiversity, worsening threats arising from land use changes and population pressure [2]. It may increase the extinction risk for endangered species [3]. Although current evidence directly linking climate change to ongoing species extinctions is limited, many studies suggest that climate change may surpass habitat loss as the primary threat to global biodiversity in the coming decades [4].

Climate change can affect the distribution and connectivity of habitats [5]. Under future climate scenarios, changes in suitable habitats are expected to intensify, causing many species to lose substantial portions of their original ranges or migrate to higher latitudes [6,7,8]. Moreover, large-scale habitat destruction and encroachment caused by human activities have made it more difficult for wildlife, especially large mammals, to expand their ranges in the context of global warming. As a result, many species have been forced to abandon their original habitats and now face an increased risk of extinction [9,10]. Species distribution models (SDMs) identify current suitable habitats, priority conservation sites, and elucidate key environmental drivers of species distributions [11]. Because of their narrow habitat tolerance, many threatened species are highly sensitive to climate change. SDMs can project future habitat suitability under various climate scenarios, highlighting regions likely to serve as refugees or at high risk of habitat loss [12]. Integrating SDMs with landscape connectivity analysis identifies potential ecological corridors, supporting population viability in the face of habitat loss, climate change, and other pressures [13].

The Amur tiger (*Panthera tigris altaica*) is a large carnivore at the top of the food chain and serves as a flagship and umbrella species for regional biodiversity conservation [14,15]. As an iconic species for ecosystem assessment and global sustainable development, it plays an irreplaceable role in maintaining healthy ecosystem functions [16]. The International Union for Conservation of Nature (IUCN), the Red Data Book of the Russian Federation, and the China Red Data Book of Endangered Animals have listed the Amur tiger as an endangered species [17,18,19]. Although the population size of the Amur tiger has increased, its survival remains uncertain [20,21]. Due to its unique geographical location, the study area provides conditions for the free movement of wildlife between China, Russia, and North Korea, maintaining critical corridors for migration and reproduction. In recent years, an increasing number of wild Amur tigers have crossed the Sino-Russian border into Chinese territory [22,23,24]. The Russian Amur tiger population is an essential source for the species’ recovery in China, and its transboundary migration helps reduce inbreeding and excessive competition [22,25,26,27]. The ecological connection between the Northeast China Tiger and Leopard National Park of China and the Land of the Leopard National Park of Russia is crucial for the survival of the Amur tiger. However, limited research has examined the impact of climate change on this species within the region [28].

Recent studies have shown that future climate change scenarios are effective tools for informing conservation planning and management [29,30]. Building on this foundation, this study aims to predict the impact of future climate change on suitable habitat and landscape connectivity of the Amur tiger. This not only supports the conservation of the Amur tiger but also enhances our understanding of how regional ecosystems respond to climate change. In addition, it provides model validation and theoretical support for China–Russia transboundary ecological corridors that have recently been established. We assume that, under climate change, the distribution of the Amur tiger will shift towards higher latitudes, resulting in corresponding changes in its suitable habitat.

## 2. Materials and Methods

### 2.1. Study Area

**Figure 1:**
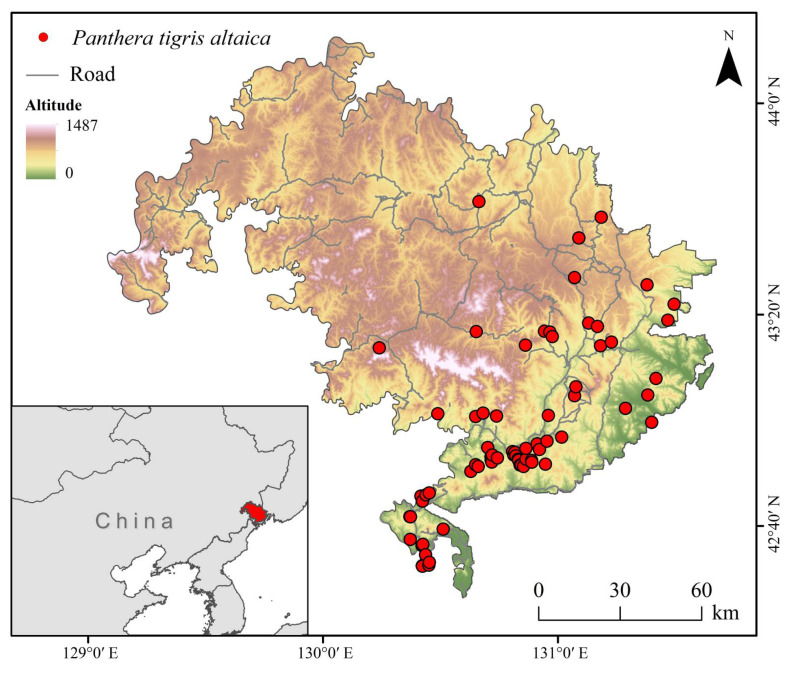
Study area and occurrence records of Amur tiger in the study area.

We selected the adjacent Northeast China Tiger and Leopard National Park and the Land of the Leopard National Park in Russia as the study area. It extends from 125°10′ E to 140°13′ E and from 41°45′ N to 53°33′ N (Figure 1), covering approximately 17,800 km^2^. The study area spans an altitudinal gradient from 0 to 1487 m, encompassing lowland plains to mid-elevation mountainous terrain. Vegetation is dominated by evergreen broadleaf forest, which accounts for 90.91% of the total area, representing a typical subtropical evergreen forest ecosystem. Other vegetation types include deciduous coniferous forest (3.43%), deciduous broadleaf forest (2.70%), dense shrubland (1.78%), and mixed forest (1.06%). The structural diversity of the vegetation provides varied ecological niches and plays a crucial role in supporting mammalian biodiversity and predator–prey dynamics in the region. The Northeast China Tiger and Leopard National Park is located in the southern part of the Laoyeling Mountains, a branch of the Changbai Mountains. The topography consists of medium to low mountains, canyons, and hills, interspersed with basins, plains, and plateaus. Elevation decreases gradually from the center outward, with valleys and low mountains in the southern and northern regions. The park experiences a temperate continental monsoon climate, characterized by windy, dry springs; short, hot summers; cool autumns with rapid temperature drops; and long, cold winters. Annual precipitation ranges from 450 to 750 mm, with approximately 80% falling between May and September. The Land of the Leopard National Park is situated in the East Manchurian Mountain region. The Borisov Plateau lies in the valley between the Razdolnaya and Amba rivers, while the Montenegrin Mountain Range is located to the south. The park falls within the Manchurian mixed forest ecoregion and experiences a temperate continental humid climate, with mild summers and cold winters. Winter monthly precipitation is often less than one-tenth of that in the wettest summer month. The average annual precipitation is approximately 826 mm, with around 80% occurring between April and October.

### 2.2. Data Collection

We collected 297 Amur tiger occurrence records from the published literature [31,32,33] and the Global Biodiversity Information Facility (GBIF; https://www.gbif.org/; accessed on 15 December 2024). To ensure the reliability of species distribution modeling, we imported species occurrence points and environmental variables into ENMTools for preprocessing and duplicate record removal based on raster resolution [11]. This step eliminated spatially redundant points and aligned the data with the environmental layers used in the analysis. In ENMTools, once species occurrence points and environmental raster data are imported, the software automatically aligns each occurrence point with the corresponding raster cell based on the standardized spatial resolution of environmental variables (30 m). If multiple occurrence points fall within the same raster cell, ENMTools treats them as duplicate records and retains only one representative point. This process removes spatial redundancy and prevents overrepresentation of specific environmental conditions in the model. After a series of filtering steps, 72 occurrence records were retained for modeling (Figure 1). The environmental variables used were obtained from the standardized data collection and processing procedures described in Section 2.3 of this study.

### 2.3. Environmental Variables

We considered four groups of environmental parameters in this study—climate, vegetation, topography, and human impact—resulting in a total of 37 environmental predictor variables (Table 1). We downloaded 19 current and future bioclimatic variables (Bio1–Bio19) from the WorldClim database (https://worldclim.org/data/index; accessed on 5 August 2024) [34]. Eight-Day Snow Cover (EDSC) and Maximum Snow Extent data (SCE) were obtained from the Level 1 and Atmosphere Archive and Distribution System (LAADS) Distributed Active Archive Center (https://ladsweb.modaps.eosdis.nasa.gov/search/; accessed on 17 August 2024). Normalized Difference Vegetation Index (NDVI), Enhanced Vegetation Index (EVI), vegetation type (Veg), and Net Primary Production (NPP) data were acquired from the Moderate Resolution Imaging Spectroradiometer (MODIS) database (https://modis.gsfc.nasa.gov; accessed on 10 August 2024) [35]. Fractional Vegetation Cover (FVC) was derived from NDVI data using spatial analysis tools in ArcGIS (v10.8.0). Digital Elevation Model (DEM) data were sourced from the Geospatial Data Cloud Platform of the Computer Network Information Center, Chinese Academy of Sciences (http://www.gscloud.cn; accessed on 3 August 2024). Slope, aspect, and altitude were calculated from the DEM in ArcGIS. Population density (POP) and Gross Domestic Product (GDP) data were downloaded from the Land Scan website (https://landscan.ornl.gov/; accessed on 11 August 2024). Human footprint (HFP) index data were obtained from the Wildlife Conservation Society (WCS) and the Center for International Earth Science Information Network (CIESIN) (https://sedac.ciesin.columbia.edu/; accessed on 22 August 2024). Grid data—including distances to the nearest settlement, man-made area, river, and road—were collected from Google Earth (https://earth.google.com/web/; accessed on 15 August 2024). Imagery was parsed, land cover patches were extracted, and classification processing was conducted in ArcGIS to refine boundaries and assign attributes.

Due to the remote location of the study area along both sides of the border, distance to the nearest settlement and the human footprint index may not adequately capture human influence. Thus, we included distance to the nearest artificial area to account for human factors more comprehensively. The climate simulations in the Coupled Model Intercomparison Project Phase 6 (CMIP6) were found to be more closely aligned with observed data. The SSP scenarios offer greater accuracy and discriminatory power and can incorporate local development factors, making them more robust than the Coupled Model Intercomparison Project Phase 5 (CMIP5) data [36]. Therefore, we selected four Shared Socioeconomic Pathways (SSP1-2.6, SSP2-4.5, SSP3-7.0, and SSP5-8.5) from the CMIP6 to represent future climate conditions in the medium term (2050s) and long term (2070s) [37].

(1) SSP5-8.5 represents a high forcing scenario characterized by rapid economic growth driven by intensive fossil fuel use and a strong emphasis on individual consumption.

(2) SSP3-7.0 is a medium-to-high forcing scenario in which the world is fragmented, international cooperation is weak, and economic development proceeds slowly.

(3) SSP2-4.5 is a medium forcing scenario that follows a continuation of historical trends, with no strong emphasis on either sustainability or fossil fuel expansion.

(4) SSP1-2.6 is a low forcing scenario representing a world with low vulnerability, low mitigation pressure, and low radiative forcing, driven by a shift toward sustainable development.

Because the primary objective of our study is to assess the impact of climate change on Amur tiger habitat, and future projections for non-climatic variables are either unavailable or incomplete, we assumed these predictor variables remain constant over time.

Since the grid data came from multiple sources and had different resolutions, we resampled the variables in ArcGIS (V 10.8.0) to a 30 m resolution. Selecting appropriate and informative environmental variables is essential for accurately assessing the effects of environmental change on species distributions. To minimize the effects of multicollinearity among variables, we used principal component analysis (PCA) to reduce dimensionality and identify representative predictors [29]. Based on pre-simulation results from the MaxEnt model, which incorporated occurrence records and environmental variables, we excluded variables with a contribution rate less than 0.1%.

### 2.4. Suitable Habitat Modeling

Many studies have used species distribution models (SDMs) to investigate the impacts of climate change on species distributions [38]. Among these, the MaxEnt model is considered one of the most effective tools for species distribution modeling. It produces robust predictions with high accuracy, even when based on limited presence data [39]. To optimize the model parameters, we adjusted the feature combinations (FCs) and regularization multipliers (RMs) to reduce the risk of overfitting. The Kuenm package in R was used to calculate the corrected Akaike Information Criterion (AICc) for various combinations of FCs and RMs, allowing us to assess model complexity [40].

We evaluated the accuracy and performance of the model predictions using the area under the receiver operating characteristic curve (AUC), the true skill statistic (TSS), and the Kappa coefficient. The AUC was calculated using the MaxEnt model, while the TSS and Kappa values were obtained using the Biomod2 package in the R (version 4.3.3) [41]. A total of 72 Amur tiger occurrence records and 15 selected environmental variables were imported into MaxEnt (version 3.4.1) for modeling. Using the optimized regularization multiplier and feature combination settings, we randomly assigned 75% of the occurrence data as training data and 25% as test data. The model was run 10 times to ensure robustness [11].

We used the jackknife test to assess the contribution of each variable, constructed response curves for the environmental predictors, and projected these relationships under future climate conditions. The results were exported in logistic format. After completing the MaxEnt model simulation, we used ArcGIS10.8 software to convert the ASCII of the Amur tiger’s suitable habitat into a raster format, where the resulting raster data represented the survival probability of the Amur tiger. We then classified the potential suitable areas using the natural breakpoint method. Based on this and incorporating the current population–environment relationship along with climate projections for the 2050s and 2070s, we predicted the future distribution of suitable habitat for the Amur tiger. ArcGIS 10.8 was used to quantify both the area and centroid position of suitable habitat in the present and future. We used the Centroid tool in ArcGIS to calculate the center point of each high-suitability area. For each period, we measured the distance and direction between centroids to analyze trends in distribution shifts.

### 2.5. Landscape Connectivity Assessment

Corridors are parts of the landscape that sustain ecological processes and enable species movement between habitats [42,43]. As species may undergo unpredictable range shifts due to climate change, it is essential to employ tools that model functional connectivity and identify ecological networks between protected areas. In this study, we used the least-cost path method for connectivity analysis, which is a widely used approach in corridor modeling [44]. Core habitats are the best habitats for wild animals, as they provide stable and sufficient food resources and hiding places. Based on the daily activity and home range of the Amur tiger’s prey, highly suitable habitat was identified and extracted using ArcGIS (V 10.8.0) to serve as core habitat patches for the Amur tiger. Based on model results and the literature, we constructed the environmental resistance surface, and weighted and superimposed various factors to develop a comprehensive resistance surface [32,45,46,47]. Ecological source areas for the Amur tiger and the corresponding resistance surface were input into the model. The Linkage Mapper (V 3.0.0) was then used with the core habitat patches to calculate cost-weighted distances and generate least-cost routes, resulting in modeled ecological corridors [48]. We used ArcGIS to calculate the number and area of core patches and extracted the number of ecological corridors from Linkage Mapper outputs. By comparing changes in these metrics across scenarios, we assessed the impact of climate on Amur tiger habitat connectivity.

## 3. Results

### 3.1. Environmental and Occurrence Data Screening and Model Setup

To minimize the effects of multicollinearity among variables, we applied principal component analysis (PCA) to reduce dimensionality and identify representative predictors (Figure S1). Combining preliminary MaxEnt simulations incorporating occurrence records and environmental variables, we excluded predictors with a contribution rate below 0.1%, retaining 15 variables for the final model (Table 1). The optimal model, identified by the lowest AICc value, used a regularization multiplier (RM) of three and a feature class (FC) combination of LQTH.

### 3.2. Model Result Accuracy

**Figure 2:**
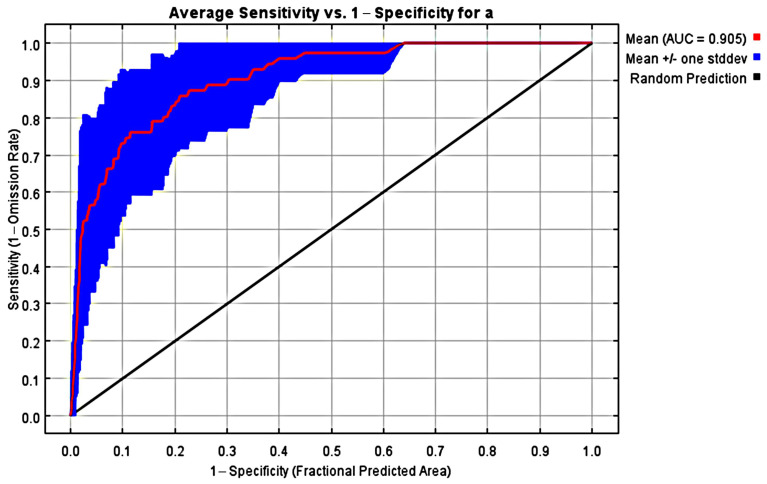
ROC curve and AUC value under the current period (10 replicated runs).

**Figure 3:**
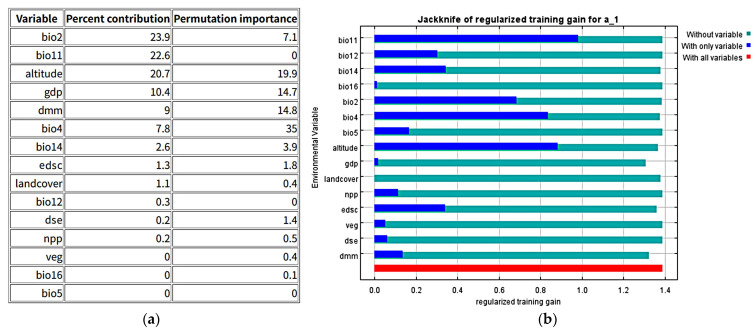
Environmental variable contribution rate and jackknife test. (**a**) Ranking of contribution rates of climate variables; (**b**) importance of various environment variables determined using the knife-cutting method.

**Figure 4:**
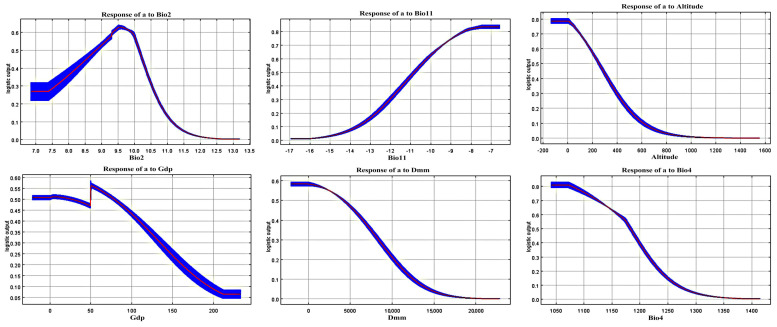
Response curves of climatic suitability for the major climate factors according to MaxEnt model (The red curves represent the mean logistic output of habitat suitability in response to each environmental variable, while the blue curves and shaded areas indicate ±1 standard deviation around the mean, reflecting model uncertainty. Each plot shows the effect of a single variable while all others are held at their average values).

The ROC analysis showed that the average AUC value for the training set was 0.905 (Figure 2), and the Kappa and TSS values calculated using Biomod2 were 0.7336 and 0.7105, respectively. These results indicate that the model’s predictions of the Amur tiger’s potential suitable habitat are highly reliable. The model identified Bio2 (mean diurnal range), Bio11 (mean temperature of the coldest quarter), altitude, GDP, and distance to the nearest man-made area (DMM) as the most important contributing variables. Additionally, Bio4 (temperature seasonality), Bio2, altitude, GDP, and DMM were key environmental factors influencing the geographic distribution of the Amur tiger (Figure 3 and Figure 4). Habitat suitability decreases significantly with increasing altitude, dropping rapidly to near zero above 800 m. The optimal habitat corresponds to an average daily temperature range of 8.70–10.15 °C. As Bio2 increases, suitability initially rises, peaking at approximately 9.5 °C before declining sharply. When the coldest month temperature (Bio11) rises from −17 °C to −7 °C, suitability rises steadily. Habitat suitability decreases as Bio4 increases. Both GDP and DMM show a significant positive correlation with the suitability of Amur tiger habitats, while negative interference is significant (Figure 4).

### 3.3. Distribution of Suitable Habitat for the Amur Tiger

**Figure 5:**
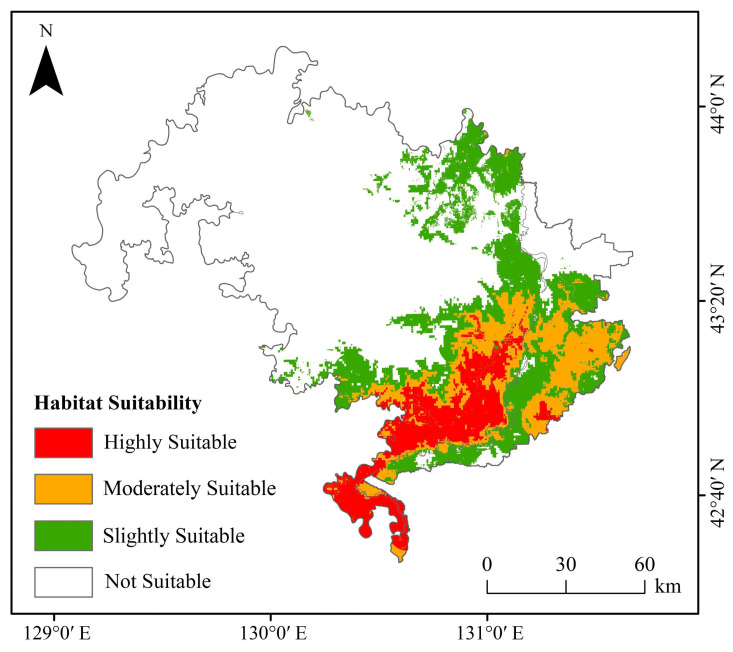
Predictions of the potentially suitable area for Amur tigers under current climate conditions based on the MaxEnt model.

**Figure 6:**
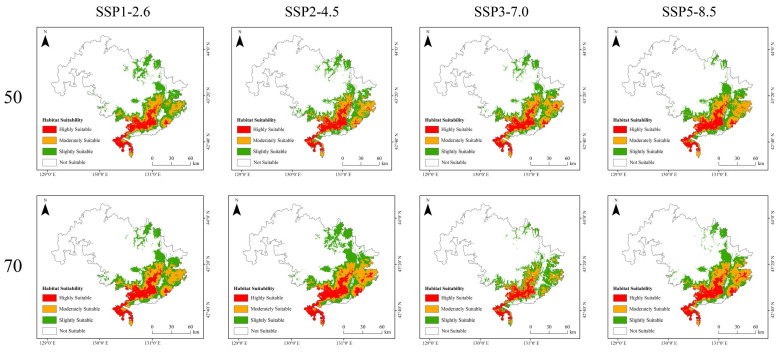
Predictions of the potentially suitable area for Amur tigers under future climate conditions based on the MaxEnt model.

Currently, suitable habitat for Amur tigers is concentrated in the southeastern part of the study area (Figure 5). Due to the influence of climate change, suitable habitats are projected to expand westward and northward by the 2050s and 2070s. The results indicated that highly suitable habitat currently covers 1147.63 km^2^ (6.42% of the study area), moderately suitable habitat covers 1580.69 km^2^ (8.84%), and slight-suitability habitat covers 2213.62 km^2^ (12.38%), while unsuitable habitat accounts for 12,926.22 km^2^ (72.34%) (Table 2). Because the study area consists of two national parks with strong conservation measures, their shared border aligns with an international boundary characterized by minimal human disturbance. As a result, highly suitable habitats are concentrated in the central portion of the study area. However, as the Amur tiger population grows, the extent of suitable habitat must expand to accommodate their needs. The highly suitable habitat comprises only a small portion of the total area and is mainly located along both sides of the Sino-Russian border. Except under the SSP3-7.0 scenario, all future climate models predict an increasing trend in suitable habitat area. By the 2070s, under SSP5-8.5, the increase in slight-suitability habitat is minimal. In contrast, the SSP2-4.5 scenario shows the most significant expansion of suitable habitat, suggesting it offers the most favorable conditions for long-term species survival. Conversely, the SSP3-7.0 scenario projects a reduction in suitable habitat, indicating that this scenario would be particularly detrimental to the future of the Amur tiger (Figure 6).

**Figure 7:**
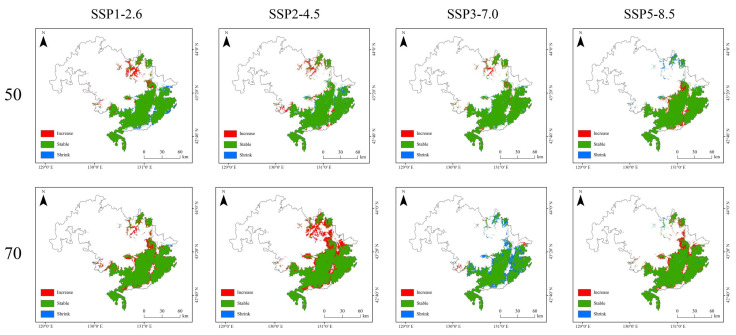
Spatial trends in the increase and decrease in suitable habitat for the Amur tiger under four Shared Socioeconomic Pathway (SSP) climate scenarios (SSP1–2.6, SSP2–4.5, SSP3–7.0, and SSP5–8.5) for the 2050s and 2070s. Red areas indicate newly gained suitable habitat, green areas represent stable suitable habitat, and blue areas denote habitat loss.

Analysis of future habitat trends under various climate scenarios revealed that changes in suitable habitat for Amur tigers were relatively minor across most models, with habitat areas largely remaining stable. Slight expansions were only observed along the edges of their current range. However, under the medium development pathway (SSP2-4.5), the suitable habitat is projected to expand considerably by the 2070s, particularly in the northern part of the study area. In contrast, under the regional rivalry scenario (SSP3-7.0), the extent of suitable habitat is expected to decline significantly by the same period (Figure 7).

**Figure 8:**
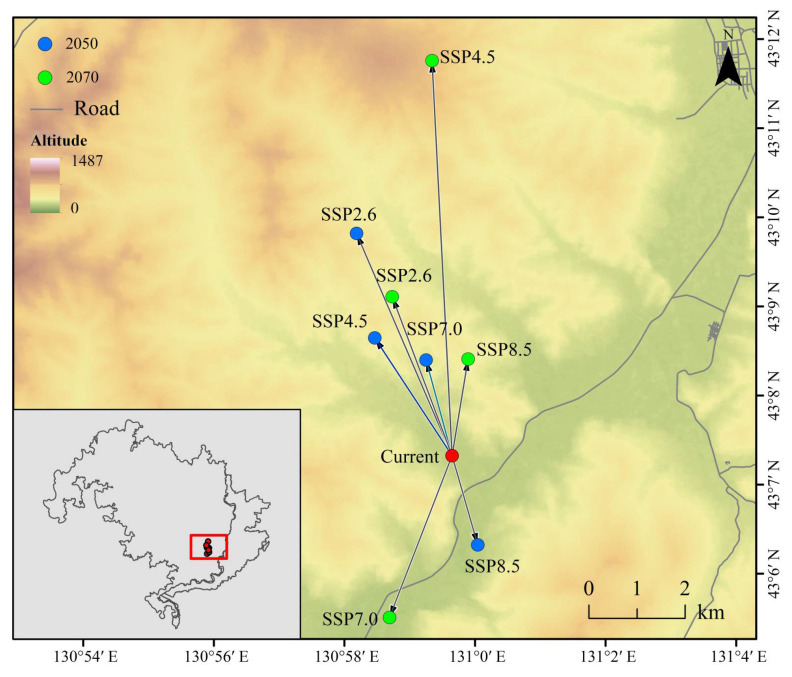
Centroid migration and change in Amur tiger high habitat area.

The centroid analysis of the Amur tiger’s highly suitable habitat under current climate conditions identified coordinates at 130°59′ E, 43°12′ N, located in the southeastern portion of the study area (Figure 8). Under most future climate scenarios, the centroid is projected to shift northward or northwestward by the 2070s. Specifically, under the medium development pathway (SSP2-4.5), the centroid is projected to move further north. In contrast, under the regional rivalry scenario (SSP3-7.0) in the 2070s and the high-emissions scenario (SSP5-8.5) in the 2050s, the centroid is anticipated to shift southward (Figure 8).

### 3.4. Current and Predicted Future Landscape Connectivity

**Figure 9:**
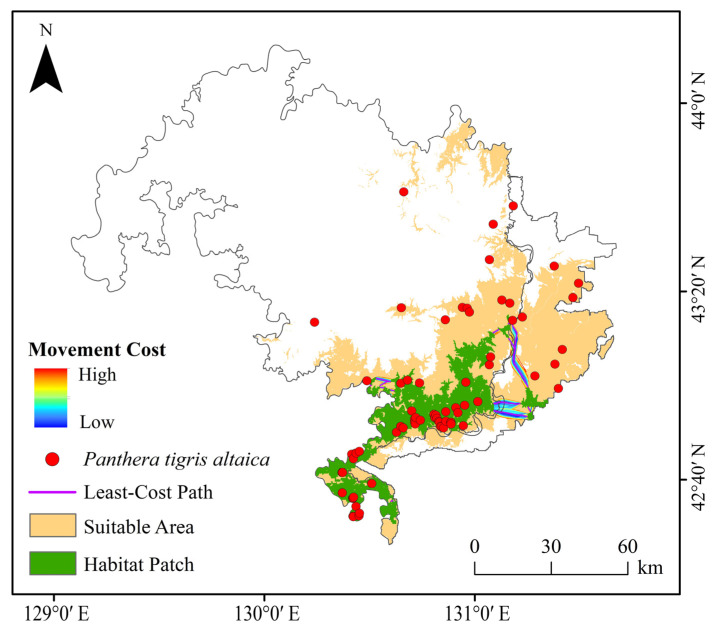
Distribution of corridors between Amur tiger habitat patches under current climatic conditions.

**Figure 10:**
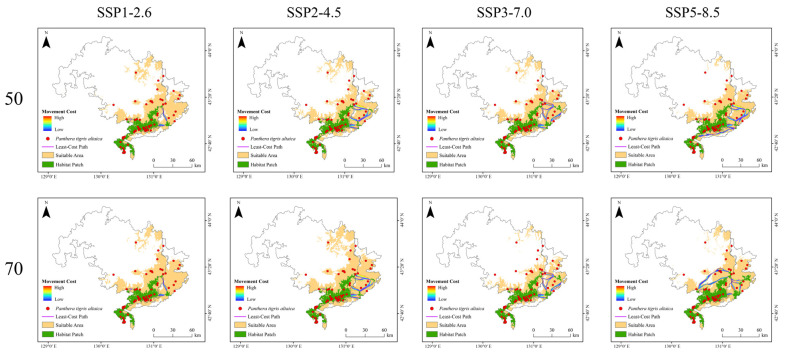
Connectivity maps among Amur tiger habitat patches under four Shared Socioeconomic Pathway (SSP) climate scenarios (SSP1–2.6, SSP2–4.5, SSP3–7.0, and SSP5–8.5) for the 2050s (top row) and 2070s (bottom row). Movement cost is shown as high (orange) or low (yellow), with suitable areas (green) and habitat patches (dark green) identified from species distribution models. Blue lines represent least-cost paths and red points indicate Amur tiger occurrence records used for validation.

Due to the clustered distribution of core habitat patches under both current and future conditions (Figure 5 and Figure 6), the number and spatial configuration of ecological corridors remain largely stable (Figure 9 and Figure 10). Under the SSP3-7.0 scenario, a significant decline in suitable habitat was accompanied by a reduction in the number of ecological corridors. In contrast, although the total habitat area under the SSP5-8.5 scenario remained stable (Table 2), the number of ecological corridors decreased to just four by the 2050s (Table 3).

## 4. Discussion

This study predicted the current and future distribution of suitable habitats and ecological corridors for Amur tigers in two adjacent national parks spanning China and Russia under multiple climate change scenarios. Results indicate that suitable habitats are shifting northward, but the habitat area shows no significant increase [49]. A limited expansion may not be sufficient to counteract the negative effects of habitat fragmentation and human disturbance on wide-ranging carnivores. These findings suggest that, even under favorable climatic conditions, the long-term viability of Amur tiger populations will depend on targeted conservation actions, including the protection of core habitats, enhancement of corridor functionality, and reduction in anthropogenic pressures.

### 4.1. Effects of Environmental Factors

As a large carnivore, the Amur tiger is influenced by a complex array of environmental and anthropogenic factors that shape its habitat suitability. Habitat use intensity decreases at elevations above 800 m, while a combination of low- and high-altitude areas may improve connectivity and enhance prey accessibility [50]. Habitat suitability patterns for Bio2, Bio4, and Bio11 suggest that Amur tigers prefer areas with high climatic stability and moderate temperature variation, whereas low temperatures and extreme fluctuations reduce suitability. GDP having a significant negative interference on suitability indicates that economic development, through urbanization and infrastructure expansion, may degrade the quality of Amur tiger habitats. Similarly, the positive correlation with DMM (distance to man-made features) implies that human disturbance contributes to habitat degradation and avoidance behavior, ultimately affecting species distribution [51,52].

### 4.2. Effects of Climate Change on Amur Tiger’s Suitable Habitat

We analyzed and simulated the spatial distribution of suitable habitat for the Amur tiger under various climate change scenarios. The results showed that habitat suitability will fluctuate depending on the scenario. Although SSP1-2.6 represents a low forcing climate scenario, it did not produce the largest increase in suitable habitat area among the four pathways. Instead, SSP2-4.5 is projected to produce the most substantial habitat gain. This suggests that factors beyond climate forcing play a critical role in shaping Amur tiger habitat expansion. The SSP3-7.0 scenario is associated with the greatest habitat loss, likely due to high population growth, regional fragmentation, and limited international cooperation [13,53]. The decline in habitat suitability in transboundary regions highlights the importance of coordinated cross-sectoral conservation efforts. Under the high-emissions SSP5-8.5 scenario, the habitat area is not expected to decrease substantially, possibly because the study area includes key protected zones that buffer against development impacts. Further model validation is needed to confirm these trends. Considering that other factors can significantly constrain habitat suitability, we need to conduct an additional simulation excluding other variables in the future.

The findings of this study aligned with previous research suggesting that the Amur tiger’s range centroid is likely to shift under future climate conditions [54]. Under projected changes in climate and land use, most species tend to migrate toward higher altitudes and latitudes; however, some may exhibit multi-directional range shifts in response to complex environmental pressures [55]. This pattern could explain the observed southward movement of the Amur tiger’s centroid under the SSP3-7.0 scenario in the 2070s and the SSP5-8.5 scenario in the 2050s.

### 4.3. Effects of Climate Change on Landscape Connectivity

Habitat changes are accompanied by transformations in the ecological corridors of the Amur tiger. Although the number of ecological corridors has varied slightly, their overall spatial distribution has remained relatively stable. Landscape connectivity analysis revealed that the model identified only a limited number of least-cost paths (LCPs), most of which were relatively long. Although the number of LCPs increased over time due to habitat changes, their spatial locations remained largely unchanged.

With rising temperatures and the continued development of transboundary ecological networks, landscape connectivity is expected to improve under future climate scenarios [56,57]. Establishing low-cost corridors between habitat patches can help reduce movement barriers, facilitate successful migration, and mitigate habitat loss driven by climate change [58,59,60]. In the future, efforts should prioritize the establishment of ecological corridors between the Sino-Russian border and fragmented patches of suitable habitat [26]. Although studies indicate that habitats along the border are highly connected, the existing literature has shown that barbed wire fences along the Sino-Russian border have hindered individual movement between the Chinese and Russian ranges of the Amur tiger [61]. This obstruction poses a significant challenge to the recovery of China’s Amur tiger population. Therefore, future conservation efforts should prioritize the removal of barbed wire fences in key ecological corridors identified by the model to enhance the functionality and effectiveness of these transboundary migration routes.

### 4.4. Implications for Conservation

Based on the findings of this study, we propose the following recommendations to support the protection and management of Amur tigers in the face of global climate change: (1) Climate change inevitably alters temperature regimes and influences both precipitation patterns and the frequency of extreme weather events. Amur tigers require habitats characterized by relatively stable temperatures and low climatic variability. Targeted conservation strategies should be implemented to mitigate or offset the adverse effects of climate change, with continued enhancement of ecological protection projects [62]. (2) Establish a long-term, systematic monitoring program to track changes in Amur tiger population dynamics and gather comprehensive data on the impacts of climate change on species distribution. (3) Based on current research, suitable habitats for Amur tigers in the study area show a westward and northward expansion trend under various climate scenarios. However, the total area has not shown a substantial increase. This suggests that other limiting factors may be constraining habitat expansion. Therefore, further efforts are needed to mitigate the negative impacts of non-climatic stressors on Amur tigers. Additionally, reports of Amur tigers entering human settlements have increased in recent years. The population along the China–Russia border remains under significant threat from human activities, which continue to restrict its range expansion into China’s interior [49]. To address this, we should expand and enhance protected areas in China, particularly in regions bordering Russia. Strategic planning should focus on creating effective buffer zones to reduce human–wildlife conflict and facilitate habitat expansion [49]. (4) Promote transboundary collaboration between China and Russia through the establishment of cross-border national parks and the development of a formal coordination mechanism. This should include regular information exchange, joint personnel training, collaborative funding efforts, and international conservation conferences. Infrastructure in shared landscapes should incorporate biodiversity-friendly designs. Conservation policies must also integrate local socioeconomic considerations to ensure the sustainable development of the region.

### 4.5. Limitations of the Method in This Article

Several limitations should be acknowledged in our modeling approach. First, variables directly reflecting land use changes were not included, which may reduce the model’s capacity to capture anthropogenic effects. Additionally, the estimates of habitat suitability and landscape connectivity were deliberately conservative to avoid overestimation [63]. Projections based on different Shared Socioeconomic Pathways (SSPs) may also fall short in representing the full complexity of future climate conditions. Future studies should aim to integrate a wider range of non-environmental variables, such as socioeconomic and land management factors, and invest in long-term, systematic field monitoring to enhance model reliability. While the occurrence data used had reasonable spatial coverage, the absence of direct field surveys may limit the accuracy of the input data. Future studies should integrate MaxEnt with complementary modeling techniques to produce more robust predictions and more reliable conservation planning outcomes. Despite these limitations, our findings offer a quantitative understanding of the projected changes in habitat suitability and landscape connectivity for the Amur tiger in the face of climate change. It provides valuable insights to inform targeted conservation strategies in the study area.

## 5. Conclusions

This study combined species niche modeling with landscape connectivity analysis to evaluate the effects of climate change on the distribution of suitable habitats for Amur tigers in the study area. The results identified the key drivers of habitat change and revealed significant variations under different climate scenarios. Projections suggest that suitable habitat areas are unlikely to expand substantially under future climate conditions. To address these challenges, conservation strategies should prioritize reducing human disturbance and implementing early warning systems and adaptive management measures to mitigate the impacts of climate change.

## Figures and Tables

**Table 1:** Environmental predictor variables considered in potential distribution modeling.

Types	Variables	Description	Units	Whether to Select
Climatic factors	Bio1	Annual Mean Temperature	°C	
	Bio2	Mean Diurnal Range (mean of monthly (max temp–min temp))	°C	√
	Bio3	Isothermality (Bio2/Bio7) (×100)	%	
	Bio4	Temperature Seasonality (standard deviation × 100)	%	√
	Bio5	Max Temperature of Warmest Month	°C	√
	Bio6	Min Temperature of Coldest Month	°C	
	Bio7	Temperature Annual Range (BIO5-BIO6)	°C	
	Bio8	Mean Temperature of Wettest Quarter	°C	
	Bio9	Mean Temperature of Driest Quarter	°C	
	Bio10	Mean Temperature of Warmest Quarter	°C	
	Bio11	Mean Temperature of Coldest Quarter	°C	√
	Bio12	Annual Precipitation	mm	√
	Bio13	Precipitation in Wettest Month	mm	
	Bio14	Precipitation in Driest Month	mm	√
	Bio15	Precipitation Seasonality (Coefficient of Variation)	%	
	Bio16	Precipitation in Wettest Quarter	mm	√
	Bio17	Precipitation in Driest Quarter	mm	
	Bio18	Precipitation in Warmest Quarter	mm	
	Bio19	Precipitation in Coldest Quarter	mm	
	EDSC	Eight_Day_Snow_Cover	-	√
	SCE	Maximum_Snow_Extent	-	
Vegetation factor	NDVI	Normalized Difference Vegetation Index	-	
	EVI	Enhanced Vegetation Index	-	
	NPP	Net Primary Production	-	√
	FVC	Fractional Vegetation Cover	-	
	Veg	Vegetation Type	-	√
	Landcover	Land Cover	-	√
Geographical factors	Altitude	Altitude	m	√
	Aspect	Aspect	-	
	Slope	Slope Degree	°	
	Dis_river	Distance from River	m	
Anthropogenic factor	Dis_road	Distance from Road	m	
	GDP	Gross Domestic Product	-	√
	Pop	The Population Density	people/km^2^	
	HFP	Human Footprint	-	
	DSE	Distance to the Nearest Settlement	m	√
	DMM	Distance to the Nearest Man-Made Area	m	√

**Table 2:** Trends of increase and decrease in suitable habitat for Amur tigers under different climate patterns in the future.

Climate Scenarios	Decade	Total Study Area (km^2^)	Total Suitable Habitat (km^2^)	Percentage of Total Area (%)	Highly Suitable Habitat (km^2^)	Percentage of Total Area (%)	Moderately Suitable Habitat (km^2^)	Percentage of Total Area (%)	Slightly Suitable Habitat (km^2^)	Percentage of total Area (%)
Current	Current	17,868.16	4941.94	27.64	1147.63	6.42	1580.69	8.84	2213.62	12.38
SSP1-2.6	2050s	17,868.16	5155.24	28.84	1115.77	6.24	1468.10	8.21	2571.37	14.39
SSP1-2.6	2070s	17,868.16	5584.52	31.24	1321.02	7.39	1723.91	9.64	2539.59	14.21
SSP2-4.5	2050s	17,868.16	5203.46	29.11	1180.33	6.60	1571.16	8.79	2451.97	13.72
SSP2-4.5	2070s	17,868.16	6275.95	35.11	1477.89	8.27	1942.10	10.86	2855.96	15.98
SSP3-7.0	2050s	17,868.16	5088.47	28.46	1166.11	6.52	1627.17	9.10	2295.19	12.84
SSP3-7.0	2070s	17,868.16	4197.24	23.48	916.79	5.13	1174.28	6.57	2106.17	11.78
SSP5-8.5	2050s	17,868.16	5075.01	28.39	1214.06	6.79	1699.67	9.51	2161.28	12.09
SSP5-8.5	2070s	17,868.16	5494.26	30.74	1383.03	7.74	1828.75	10.23	2282.48	12.77

**Table 3:** Core patches and corridor properties for the Amur tiger across climate change scenarios.

Climate Scenarios	Time Period	No. of Core Patches	Total Area of Core Patches (km^2^)	Area of Largest Core Patches (km^2^)	No. of LCPs
Current	Current	10	1122.04	769.62	15
SSP1-2.6	2050s	5	1095.59	1043.33	6
SSP1-2.6	2070s	6	1308.25	1223.90	8
SSP2-4.5	2050s	11	1150.66	804.17	17
SSP2-4.5	2070s	9	1461.97	1257.19	14
SSP3-7.0	2050s	11	1141.84	693.37	19
SSP3-7.0	2070s	6	887.05	254.48	6
SSP5-8.5	2050s	4	1191.38	1123.79	4
SSP5-8.5	2070s	6	1353.77	1161.66	10

## Data Availability

The data presented in this study are available on request from the corresponding author.

## Supplementary Materials

The following supporting information can be downloaded at: https://www.mdpi.com/article/10.3390/ani15172466/s1, Figure S1: Principal component analysis (PCA) analysis screening of environmental factors results.
