# Supplementary figures and images for: Impacts of Climate Change on Habitat Suitability and Landscape Connectivity of the Amur Tiger in the Sino-Russian Transboundary Region
Source: Animals (Basel). 2025 Aug 22;15(17):2466. doi: 10.3390/ani15172466 (PMC12427190; doi:10.3390/ani15172466)

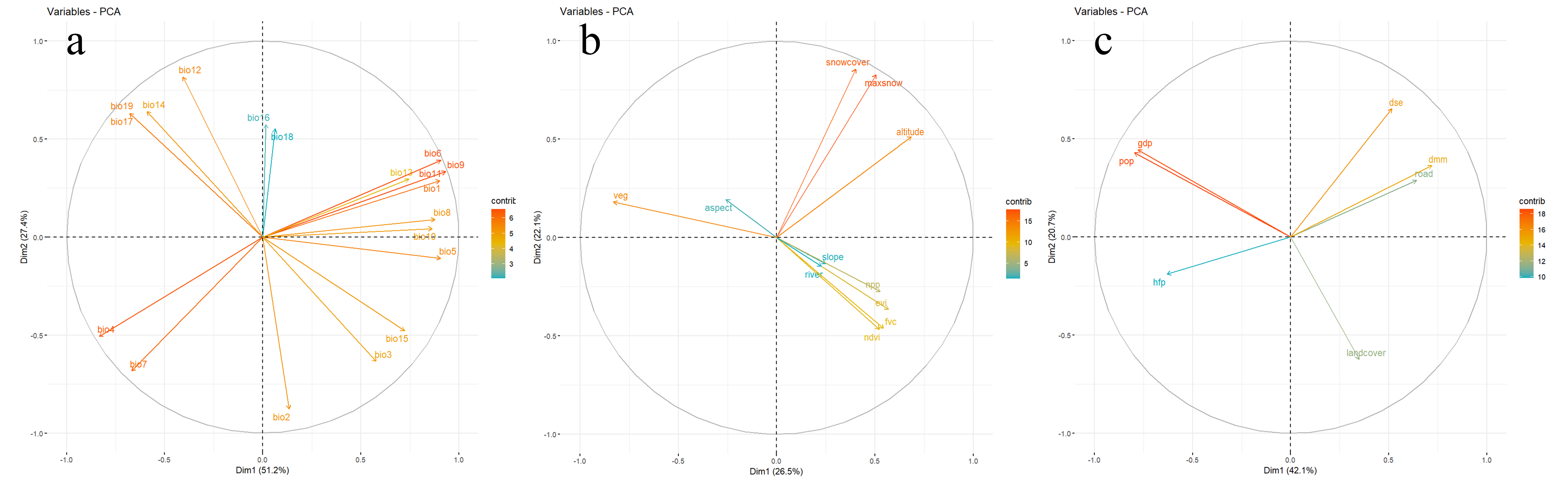

Supplement: Supplementary file 1 [file animals-15-02466-s001.zip › animals-3795184-supplementary.jpg]
